# Sire and liveweight affect feed intake and methane emissions of sheep confined in respiration chambers

**DOI:** 10.1017/S1751731114001773

**Published:** 2014-08-04

**Authors:** D. L. Robinson, J. P. Goopy, A. J. Donaldson, R. T. Woodgate, V. H. Oddy, R. S. Hegarty

**Affiliations:** 1 CRC for Sheep Industry Innovation, University of New England, Armidale, NSW 2351, Australia; 2 NSW Department of Primary Industries, Beef Industry Centre, University of New England, Armidale, NSW 2351, Australia; 3 University of New England, Armidale, NSW 2351, Australia

**Keywords:** sheep, feed intake, methane, genetics, bias

## Abstract

Daily methane production and feed intake were measured on 160 adult ewes, which were the progeny of 20 sires and 3 sire types (Merino, dual-purpose and terminal) from a genetically diverse flock. All animals were housed in individual pens and fed a 50/50 mix of chaffed lucerne and oaten hays at 20 g/kg liveweight (LW), with feed refusals measured for at least 10 days before the first of three 22-h measurements in respiration chambers (RC). Feed was withdrawn at 1600 h on the day before each RC test to encourage the ewes to eat the entire ration provided for them in the RC. After the first 1-day RC test, the sheep were returned to their pens for a day, then given a second 1-day RC test, followed by another day in their pens, then a third RC test. After all animals had been tested, they were ranked according to methane emissions adjusted for feed intake in the RC and on the previous day, enabling 10 low and 10 high methane animals to be chosen for repeat measurement. No variation between sires nor consistent effects of LW on feed eaten (%FE, expressed as per cent of feed offered) was evident in the 10 days before the first RC measurement. However, significant differences between sires (equivalent to an estimated heritability of 41%) were identified for %FE during the 2^nd^ and 3^rd^ days of RC testing (2 and 4 days after the initial RC test). The analysis of all data showed that methane emissions in the RC were related to feed intake on the day of testing and the two previous days (all *P*<0.0005). Before correcting for feed intake on previous days, there was some variation between sires in methane yield, equivalent to an estimated heritability of 9%. Correction for feed intake on the 2 previous days halved the residual variation, allowing other effects to be detected, including effects of LW, twins reared as singles, test batch, RC and test-day effects, but estimated sire variation fell to zero. In order to avoid potential biases, statistical models of methane emissions in the RC need to consider potential confounding factors, such as those identified as significant in this study.

## Implications

Despite a long adaptation to their pens and dietary environment, when confined in respiration chambers (RC) for methane measurement, both sire and liveweight (LW) affected the willingness of sheep to consume their daily feed ration. Methane emissions were significantly related to feed eaten in the RC and 2 previous days; accounting for these relationships reduced sire variation in methane yield from a small positive value to zero. Future analyses should consider fitting covariates for feed eaten on previous days, the effect of confinement in the RC on animal behaviour, and effects of environmental factors such as twins reared as singles.

## Introduction

Enteric methane (CH_4_) emissions of livestock represent a substantial proportion of greenhouse gas emissions from agriculture – in Australia, 68% of all agricultural emissions (Department of Climate Change and Energy Efficiency (DCCEE), 2012). Global demand for meat and milk is predicted to double over the period 2000 to 2050 (Gerber *et al.*, 2010). It will be a challenge to meet this anticipated increase in production without increasing emissions of the greenhouse gas, methane, from ruminant livestock.

One possibility is to breed animals for reduced methane emissions (Hegarty and McEwan, 2010; Wall *et al.*, 2010). Respiration chambers (RC) under controlled conditions are often considered to be the ‘gold standard’ way of measuring ruminant methane emissions. Animals are commonly fed at a fixed proportion of maintenance requirements, and the results expressed as methane yield (MY), calculated as daily methane production (DMP, g CH_4_/day), divided by feed intake while in the RC (Pinares-Patiño *et al.*, 2011b).

However, CH_4_ emissions of sheep are dependent not just on feed intake in the RC, but also on feed eaten on previous days (Bickell *et al.*, 2011). In addition, confinement in the RC has been shown to influence the amount of feed eaten when animals have *ad libitum* access to feed (Robinson *et al.*, 2011; Freetly and Brown-Brandl, 2013). Here we examine sire variation and phenotypic relationships for RC measurements of methane emissions and feed intake of sheep habituated to a diet of lucerne and oaten chaff, fed at a restricted rate of 20 g/kg liveweight (LW).

## Material and methods

All animal procedures were carried out under ACEC approval number UNE 09/144.

### Selection of sheep and measurement of methane emissions

As described by Goopy *et al.* (2014b), a total of 800 1-h methane measurements were recorded on 710 non-pregnant, non-lactating mature ewes (born in 2006) in July 2009 at the Falkiner Memorial Field Station at Deniliquin, NSW, Australia. The ewes belonged to the SheepGENOMICS flock, which was created to provide a high level of genetic diversity to facilitate gene discovery (White *et al.*, 2012). Over a period of 12 days, methane emissions were recorded in the field by testing batches of 15 sheep in individual portable accumulation chambers (PAC; Goopy *et al.*, 2011) for 1 h at 0900, 1030, 1200, 1330 and 1500 h (a total of 75 sheep/day). The protocol included an overnight fast, which continued until 2 h before measurement, at which time the animals were given access to baled wheaten hay (90% dry matter (DM); 8.6% CP and 9.1 MJ ME/kg DM) for 1 h. The 1-h methane measurement commenced 60 min after the end of the feeding opportunity (Robinson *et al.*, 2010).

This measurement was used to select 207 sheep with low (*n*=104) or high (*n*=103) methane emissions (adjusted for LW) for transfer to the NSW Department of Primary Industries Glen Innes Agricultural Research Station where they were remeasured under the same protocol. Some weeks later, in May 2010, the sheep were measured twice under a different protocol of rounding up from pasture all the animals to be tested that day and putting them into a holding paddock (with a limited amount of feed) before the first test. Batches of 14 sheep were tested for 1 h in PACs in sessions at 0900, 1030, 1200, 1330 and 1500 h. On the basis of these measurements, a subset of 160 ewes representing divergence in methane emissions adjusted for LW were selected for measurement in RC. The ewes were offspring of 20 sires (12 Merino (M), 4 terminal (T) and 4 dual-purpose (DP) sires) mated to purebred Merino or Merino×Border Leicester (M×BL) dams. Numbers of offspring by sire type, dam breed and birth and rearing types are shown in Table 1.

**Table 1:** Number of animals by sire type (DP, M and T), dam type (Merino or Merino×BL), birth and rearing type

	Sire type^1^		
	DP	M	T
Number of sires	4	12	4
Offspring per sire (mean)	6.8	8.6	7.0
Offspring per sire (minimum, maximum)	4, 10	5, 14	4, 9
Offspring (Merino dams)	12	103	12
Offspring (Merino×BL dams)	13	0	13
Offspring (unknown dam type)	2	0	3
Born and reared as singles (%)	44	68	37
Born and reared as twins/multiples (%)	40	18	41
Multiple birth, reared as single (%)	16	15	22

DP=dual-purpose; M=Merino; T=Terminal; BL=Border Leicester.

1 Two animals had unknown sires and sire types.

Initial RC measurements were carried out in four batches of 40 animals: RC1, 21 June to 2 July and 12 to 23 July 2010; RC2, 16 to 27 August and 6 to 7 September 2010; RC3, 4 to 15 October and 8 to 19 November 2010; RC4, 14 to 25 February and 28 February to 11 March 2011. The batches of sheep were transported from Glen Innes to the University of New England, Armidale, where they were shorn before being housed in individual pens and adapted over a 2-week period to a 50/50 mix of lucerne and oaten chaff (Manuka Feeds Pty Ltd, Quirindi, NSW, Australia; 88.5% DM; 167 g CP/kg DM, 8.7 MJ ME/kg DM) fed at 20 g/kg LW as a single meal at 0800 h each day. Feed refusals were recorded for 10 days before the first RC test (day 0). To encourage animals to eat their full ration in the RC, feed was withdrawn at 1600 h the day before each RC test.

As described by Bird *et al.* (2008), air flow through each chamber (mean=98.8 l/min) was measured using an AL800 dry gas meter (American Metering Company, Nebraska City, NE, USA) and CH_4_ concentration (ppmv) measured in incoming and exhaust air streams using an Innova 1312 Multigas Analyser (California Analytical Instruments, Orange, CA, USA). Air temperature and pressure were measured by Easysense sensors (part nos. 1113201, 113220, 113264; Serrata Pty Ltd, Sydney, Australia) to enable DMP (g/day) to be calculated.

Feed refusals were measured at the end of the 22-h CH_4_ measurement period, then animals returned to their pens for a day, after which CH_4_ emissions were again measured in the RC (day 2), with a third 22-h RC measurement on each animal on day 4.

### Repeat measurements of 20 extreme animals

Emissions data were used to calculate MY (i.e. methane emissions divided by feed intake in the RC) from all records where the sheep ate at least 75% of the feed provided in the RC. In addition, a model including LW, chamber, batch, batch×chamber, feed intake in the RC and on the previous day was fitted to emissions data. The results were used to identify a subset of 10 high and 10 low emitters for further study in test batch 5 (March/April 2011), with four additional RC measurements per animal (Goopy *et al.*, 2014a). Feed intake of these animals was recorded for 15 days before the first two RC measurements in batch 5 (days 0 and 2), which used a similar protocol to the initial measurements. The third and fourth RC measurements took place on days 12 and 14, after an interval of 6 days (either days 5 to 10 or 6to 11) when the sheep were housed in metabolic crates to enable faeces and urine to be collected.

### Statistical analyses

Analyses were carried out using R software (R Development Core Team, 2005), fitting linear mixed models using the REML methodology (Robinson, 1987) in ASREML-R (Butler *et al.*, 2009).

#### LW

The variation in LW from May 2010 (when all animals had 1-h PAC tests), over the course of the five RC test batches (June 2010 to April 2011) was examined by fitting a mixed linear model:(1)
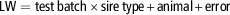
where animal was fitted as random, and test batch×sire type was a fixed effect representing the interaction of test batch (six levels, comprising the PAC test in May 2010, plus batches 1 to 5 in the RC) and sire type (Merinos or dual-purpose/terminal (DP/T); the latter had very similar changes in weight over time, so were combined into a single category).

LW in the RC (LWRC) was also regressed directly on LW in May 2010 by fitting the model:(2)
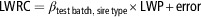
for regression coefficients, *β*_test batch×sire type_, of LWRC on LW of the same animal in the paddock (LWP) in May 2010, with different coefficients for each test batch and the two main sire types (M and DP/T). In this model, the intercepts were not significantly different from zero, so were omitted.

#### Feed eaten before and during RC tests

Sire variation in feed eaten (%FE) as per cent of feed offered, for each day from 10 days before the first RC measurement to the end of testing, was examined by fitting separate models for each individual day:(3)
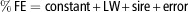
where sire was fitted as random and %FE was for a particular test day, for example, day 1.

#### DMP v. feed intake

The relationship between DMP and feed intake was examined (in a combined analysis of all test days) by fitting the model:(4)
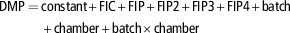
where FIC is the feed intake in the RC and FIP, FIP2, FIP3 and FIP4 the daily feed intake (weight of feed eaten, not DM intake), respectively, 1 to 4 days before testing. All terms except FIP3 and FIP4 were highly significant (*P*<0.0005) with FIC explaining 66.6% of the variation. Adding FIP+FIP2 increased the multiple *R* ^2^ to 69.9%, with the full model including random effects of batch, chamber and batch×chamber (but omitting FIP3 and FIP4) explaining 76.6%.

Two variants of MY were calculated:(5a)
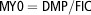
where FIC=feed intake in the chamber, and(5b)
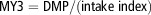
for intake index=0.509×FIC+0.342×FIP+0.148×FIP2 where the estimates of the relative weights for FIC, FIP and FIP were derived from equation 4.

#### Repeated-measures analysis of RC measurements

A combined analysis was conducted of all DMP measurements to estimate the variation within and between animals and sires (pedigree information was not available for sires). Terms considered for inclusion in the models were: LW, feed intake, birth and rearing types (single or twin/multiple), TwinRS (an indicator variable, equal to 1 if the animal was from a twin or multiple birth but reared as a single, and 0 in all other cases), sire type (sire type – D, M or T), dam breed (dam breed – M or M×BL), sire (the animal’s sire), animal, batch (the test batch of the animal – June/July, August/September, October/November, February/March, March/April), chamber (the RC, 1 to 5, in which the measurements were made) and dayno – a factor representing the day number of the RC test (0, 2, 4, 12 or 14 days after the first RC measurement).

The final model, fitted to the traits of DMP and MY0 was(6)
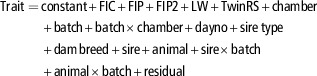
where FIC, FIP and FIP2 and the other terms in the model are described above. In this analysis, the first six terms were fitted as fixed, the remaining terms as random.

A model adjusting for LW, but not feed intake, was also fitted to the traits of %FE, DMP, MY0 and MY3:(7)
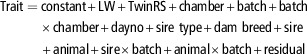
Finally, to estimate the effect of the different test batches on feed intake in the RC, including differences related to variation in the weight of animals tested at different times of year, equation (7) omitting the covariate for LW, was fitted to all data for feed intake (g/kg LW) in the RC.

#### Repeatabilities

Repeatability (correlation between repeat measurements) of 22-h measurements of the same animal in different batches was estimated as rpt=*V*_aa_/*V*_T_, where *V*_aa_ is the estimated variation due to all animal effects (the sum of variation due to animals, their sires, sire types and dam breeds) and *V*_T_ the total variation of animal effects (*V*_aa_+*V*_r_+*V*_sb_+*V*_ab_, where *V*_r_ is the residual variation, *V*_sb_ the sire×batch interaction, *V*_ab_ the animal×batch interaction). For repeat tests of animals in the same batch, sire×batch and animal×batch effects are unchanged, so repeatability of measurements on the same animal in the same batch was estimated as (*V*_aa_+*V*_sb_+*V*_ab_)/*V*_T_.

## Results

### Test batch effects on LW and FI

Animals were offered feed as a fixed proportion of LW. Variation in LW therefore affected the amount of feed provided, leading to variation in feed intake, which contributed to the differences in methane emissions of animals tested in different batches. Table 2 shows that sheep tested in late winter and early spring (August/September and October/November) had the lowest LW. Merinos were, on average, lighter than offspring of terminal and dual-purpose sires, and ate a higher proportion of their ration in the RC (19.1 g/kg LW compared with 18.1 g/kg LW for non-Merinos, *P*<0.02). However, test session effects on feed intake, expressed as g/kg LW, were relatively minor (Merinos, 18.9 to 19.3 g/kg LW; non-Merinos 17.6 to 18.6 g/kg LW).

**Table 2:** Number of sheep tested, predicted means for liveweight by sire type (Merino or terminal/dual-purpose) in May 2010 (at pasture, P0) and RC test batches 1 to 5, plus predicted means for feed intake in the RC by test batch and sire type

	P0^1^	RC1	RC2	RC3	RC4	RC5	Average	Sire type
Test batch	May 2010	June/July	August/September	October/November	February/March	April 2011	s.e.d.^2^	means
No. of sheep	160	40	40	40	40	20		
Liveweight (kg)								
Non-Merino	81.6	70.5^a^	69.1^b^	67.7^c^	74.8^bd^	77.9^e^	1.29	73.6^A^
Merino	66.1^ad^	54.9^e^	50.6^f^	50.9^c^	54.3^f^	53.8^c^	0.79	55.1^B^
Test batch means	73.9	62.7	59.8^a^	59.3^a^	64.5^b^	65.9^b^	0.76	
RC weight, per cent of May 2010 weight								
Non-Merino		86.6^a^	84.6^ab^	82.5^bc^	91.5	96.5	1.63	88.3^A^
Merino		82.8^bd^	76.3^e^	77.0^e^	82.4^bf^	81.3^cdf^	1.18	80.0^B^
Test batch means		84.7^a^	80.4^b^	79.7^b^	87.0^c^	88.9^ac^	0.99	
Feed intake in the RC (g/kg liveweight)								
Non-Merino		18.4^a^	18.1^ab^	18.6^a^	17.6^bc^	18.0^ac^	0.48	18.1^a^
Merino		18.9^a^	19.2^a^	19.3^a^	18.9^a^	19.2^a^	0.39	19.1^b^
Test batch means		18.7^a^	18.7^a^	19.0^a^	18.2^a^	18.6^a^	0.27	

RC=respiration chamber; PAC=portable accumulation chamber. ^a–f^^,^^A, B^For each set of means (test batch means, sire type means, sire type×batch means) those without a common lowercase superscript (including means without any superscripts) differ (*P*<0.05). Sire type means with different uppercase superscripts differ (*P*<0.0001).

1 Weight when all sheep were at pasture and had 1-h PAC measurements.

2 Average s.e. of differences between means in the same row for test batches (P0, RC1 to RC5).

### %FE: first four batches

As noted earlier, the results of fitting equation (4) showed that methane emissions were significantly related to feed intake in the RC and previous 2 days. Knowledge of the variation in feed intake is therefore important in helping to understand the variation in DMP.

**Figure 1:**
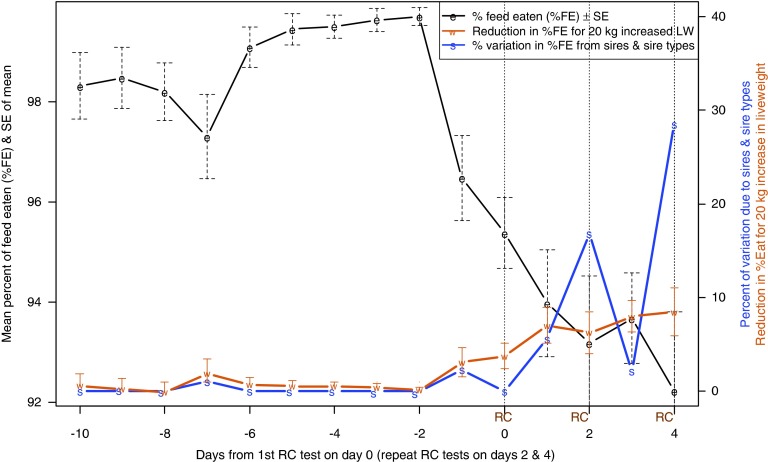
In batches 1 to 4, variation over time in the percentage of offered feed that was eaten (%FE, means±s.e.), reduction in %FE (means±s.e., right-hand axis) for 20 kg increased live weight (LW) and percentage of variation in %FE attributed to the animals’ sires (right-hand axis).

Predicted means (±s.e. from equation (3)) for %FE over the course of the test procedure are shown in Figure 1, where dashed vertical lines indicate RC test days. From 10 to 7 days before their first RC test, when still adjusting to their surroundings, average feed intake of the ewes was about 98% of the supplied ration (Figure 1). Variation in %FE reduced markedly thereafter, with %FE increasing to a peak of 99.7% 2 days before the first RC measurement. By the time feed was withdrawn at 1600 h on the day before the first RC measurement, the ewes had eaten 96.5% of their ration. There was a gradual decline in average %FE over the next 5 days as the sheep underwent a day of RC testing, a day in the pen, another day in the RC, another day in the pen, until, on the final day of RC testing, the ewes ate an average of 92.2% of feed on offer.

#### Relationships between %FE and LW by test day

The effect on %FE of being 20 kg heavier (±s.e., from equation (3)) is also shown in Figure 1. Even though the ration of 20 g feed/kg LW represents a slightly greater proportion of maintenance requirements for heavier sheep, the effect of LW was not significantly different from zero until the day before RC testing, when heavier animals started to eat a lower proportion of their ration. The effect increased in magnitude until the final day of RC testing when a sheep 10 kg heavier than average was estimated to eat 87.9% of its ration, compared with 96.4% for a sheep 10 kg lighter than average.

#### Sire variation in %FE by test day

As with LW, virtually no variation in %FE was explained by the animals’ sires until the start of RC testing, when the proportion of variance (over and above that explained by LW) started to increase, reaching a peak on the last day of testing (Figure 1). Significant differences between sires were noted on days 2 (*P*=0.044) and 4 (*P*=0.013), corresponding to the second and third time animals were measured in the RC. This indicates that there is a genetic component to the reduction in %FE in response to confinement in the RC for methane testing. In contrast, from 10 to 7 days before the first RC test, when the sheep may have been adapting to the change from grazing pasture to individual pens and a feeding regime of 20 g/kg LW, the ewes ate a lower proportion of the feed offered, but there were no significant differences between sires in %FE (Figure 1).

### %FE: repeat test on 10 high and 10 low emitters

**Figure 2:**
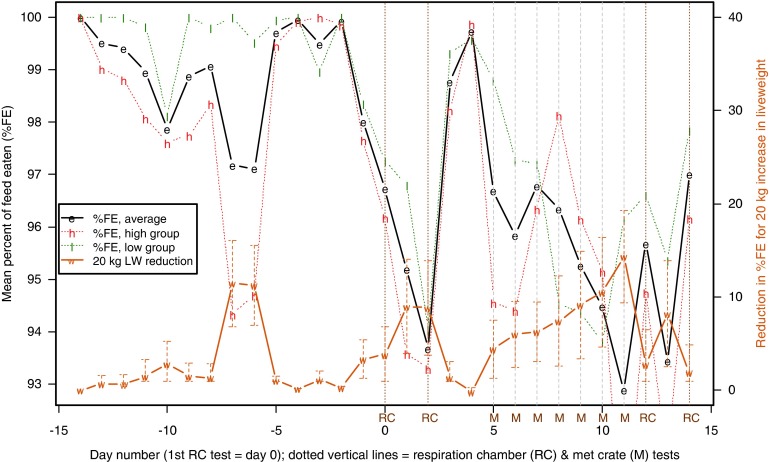
Variation over time in batch 5 in the percentage of feed offered that was eaten (%FE) for the 10 high and 10 low emitters (average for high and low groups, and all 20 sheep) and the effect on %FE of 20 kg increased liveweight (LW, means±s.e., scale on right-hand axis). Respiration chamber testing was on days 0, 2, 12 and 14; confinement in metabolic crates (M) was either days 5 to 10 or days 6 to 11.

The test protocol for the fifth batch, measuring methane from 10 high and 10 low emitters was somewhat different, with 4 days of RC testing plus 6 days (either days 5 to 10 or 6 to 11) in metabolic crates (Figure 2). Although means for 10 animals are more variable than the means for all 160 sheep in Figure 1, feed intake was again depressed during the first 2 days of RC testing, recovered when the animals were returned to their pens for 2 or 3 days, then declined for the 6 days in metabolic crates and the final 2 days of RC testing. Heavier animals showed greater reductions in %FE than lighter ones.

### Sire and phenotypic variation of RC measurements adjusted for LW

Table 3 shows the results from fitting equation (7) (fitting LW but not feed intake) to %FE, DMP, MY0 and MY3, as well as equation (6) to DMP and MY0. Consistent with Figures 1 and 2, which show greater variation in %FE for the second and subsequent RC tests, although sire variation in %FE was not significantly different from zero in the analysis of all data (*P*=0.18), the analysis restricting %FE to the 2^nd^ and 3^rd^ days of RC testing revealed greater variation between sires (significantly different from zero, *P*<0.05) equivalent to an estimated heritability of 41%. This suggests that the variation in %FE inherited from the animals’ sires is more likely to be expressed after repeated days of RC testing.

**Table 3:** Means, estimates of variation due to sires, animals, test batches and RC, repeatabilties (of tests in the same and different batches) plus effects of increased LW and rearing twins as singles on methane and feed intake traits: per cent of offered feed that was eaten (%FE), DMP and methane yield (calculated by ignoring (MY0^1^) and accounting for (MY0^2^) feed eaten before the day of testing)

	%FE all data	%FE (2 and 4 days after first RC test)	DMP CH_4_ (g/day)	DMP^3^ CH_4_ (g/day) covs for FI	MY0^1^ (g/kg FIC)	MY0^3^ ^,^ ^4^ (g/kg FIC), covs for FI	MY3^2^ (g/kg FI)
Mean	94.1	93.0	22.2	22.2	20.2	20.2	19.9
Repeatability (same batch)	34.3%	44.1%	52.0%	39.8%	22.9%	36.2%	41.0%
Repeatability (different batch)	20.6%	10.2%	23.2%	27.5%	21.8%	24.9%	27.5%
CV (sire variation)^5^	2.5%	4.4%	2.8%	0	2.0%	0	0.0%
CV (total animal variation)^6^	5.6%	4.4%	6.4%	5.1%	6.2%	5.1%	5.0%
Estimated variation due to sire and animal effects and interactions							
Sires±s.e.	5.4±5.3	16.9*±11.1	0.39±0.38	0.00	0.16±0.32	0.00	0.00
*P*-value (sire variation=0)	0.183	0.046	0.240	0.997	0.460	0.998	1.000
Animals within sires	22.0	0.0	1.65	1.22***	1.39*	1.05*	0.98***
Sires×batch	2.1	0.0	0.00	0.25	0.08	0.36	0.35
Animals×batch	16.2	56.5	2.54*	0.31	0.00	0.12	0.13
Residual	87.5	93.0	4.24	2.75	5.61	2.71	2.13
Effect of LW (per 10 kg increase) and twins/multiples reared as singles (TwinRS)							
LW±s.e.	−2.73***±0.71	−3.91***±1.00	2.85***±0.21	−0.05±0.22	−0.20±0.17	−0.56**±0.20	−0.28*±0.12
TwinRS±s.e.	2.87±1.78	3.82±2.2	1.30**±0.51	1.02**±0.35	0.22±0.39	0.86**±0.32	0.80**±0.30
Estimated variation due to test batch, chamber and test day							
RC test day (1^st^, 2^nd^, etc. )	1.20	0.00	0.16*	0.13	0.14	0.05	0.12*
Test batch	0.00	0.81	0.90*	0.58*	0.60**	0.60**	0.45*
Chamber	0.41	1.32	0.67*	0.54*	0.25	0.40	0.41*
Chamber×batch	0.00	0.00	0.60***	0.47***	0.20	0.31	0.35***

RC=respiration chamber; DMP=daily methane production; FI=feed intake; LW=liveweight.

1 MY0=DMP divided by feed intake in the RC.

2 MY3=DMP/(intake index) where intake index=0.509×FIC+0.342×FIP+0.148×FIP2 (equation (3)), where FIC is the feed eaten in the RC (kg), FIP the feed eaten on the previous day and FIP2 the feed eaten 2 days before testing. All feed measurements were weights of feed eaten (i.e. not dry matter intake).

3 Analysis of DMP with covariates for FIC (estimated coefficient: 9.06***±0.65), FIP (6.74***±0.82) and FIP2 (2.46**±0.79).

4 Analysis of MY0 with covariates for FIC (−13.75***±0.64), FIP (5.86***±0.79) and FIP2 (2.82***±0.77).

5 CV (sire variation)=sqrt(sire variation)/trait mean.

6 CV (total animal variation)=sqrt(variation due to animals, sires, sire types and dam breeds)/trait mean.

*^,^ **^,^ ***Significantly greater than zero, *P*<0.05 or *P*<0.01, *P*<0.001, respectively.

In contrast to the significant sire variation for %FE, estimated sire variation for DMP and MY0 from fitting equation (7) (omitting covariates for feed intake) was quite small and not significantly different from zero (*P*=0.24 and 0.46, respectively, equivalent to estimated heritabilities of 18% for DMP and 9% for MY0). When FIC, FIP and FIP2 were included in the model, estimated sire variation fell to zero. For DMP, the regression coefficients were all positive (9.06***±0.65 for FIC, 6.74***±0.82 for FIP and 2.46**±0.79 for FIP2). For MY0, the coefficients were −13.75***±0.64 for FIC, 5.86***±0.79 for FIP and 2.82***±0.77 for FIP2. The highly significant negative coefficient for FIC strongly suggests that using MY0 can ‘over-adjust’ for FIC, and that the relatively small estimate of sire variation for MY0 was due to the offspring of some sires being more likely to have reduced feed intake when repeatedly confined in the RC. For MY3, which fully accounts for feed intake in the RC and previous 2 days, estimated sire variation was zero.

Trait MY3, which accounts for the variation due to feed intake on previous days, had residual variation of 2.13, less than half the residual variation of 5.61 for MY0 (Table 3). The smaller residual variation of MY3 improved the ability of the models to detect systematic effects. For example, twins reared as singles had greater MY3 (0.80±30, *P*<0.01) than twins reared as twins, or singles reared as singles, but the estimate of the same effect for MY0 was not significantly different from zero. Similarly, perhaps because of the higher residual variation, no significant effect of LW could be detected on MY0, but a significant effect of −0.28±012 (*P*<0.01; Table 3) for a 10 kg increase in LW was noted for MY3.

MY0 is a difficult trait to interpret and understand, because it ignores the effect on DMP of feed eaten on the 2 previous days. At the phenotypic level, there was a moderately strong correlation between MY0 and %FE (*r*=−0.46), implying that animals that ate a higher proportion of their ration in the RC had lower MY0. Feed intake (kg) in the RC was also negatively correlated with MY0 (*r*=−0.36). Trait MY3, which accounts for feed intake on previous days, had much lower phenotypic correlations (*r*=−0.09, for feed eaten in the RC and *r*=0.01, for %FE) suggesting that phenotypic selection for MY3 would be less likely to affect feed intake than phenotypic selection for MY0.

To explore potential biases in MY0 from the impact of repeated confinement in the RC, a separate analysis was also conducted of MY0, restricted to the first RC measurement (day 0), fitting equation (6) (including covariates for feed intake) but omitting the animal×batch interaction (which represent the residual variation when there is only one measurement per animal in each batch). The residual variation was 2.08, somewhat lower than for the analysis of all test days (2.75), with significant regression coefficients for FIC (−9.63***±1.37) and FIP (6.67***±1.40) and estimated sire variation of 1.40±2.25 (equivalent to an estimated heritability of 0.16±0.26).

Estimates of the repeatability of tests in the same batch (i.e. tests conducted a few days apart) are provided in Table 3, together with repeatabilities for tests in different batches, estimated in the modelling process mainly by comparisons of the 20 animals re-tested in batch 5 with previous results. All traits had relatively low repeatabilities for measurements in different batches (21% to 27%). For the traits shown in Table 3, expressing the total s.d. of animal variation (including any due to sires and sire type) as a percentage of the mean, resulted in CVs of animal effects ranging from 4.4% (%FE on days 2 and 4) to 6.4% (DMP ignoring feed intake).

## Discussion

### Repeatability and heritability of MY

Our results confirm those of other researchers that RC measurements of MY have relatively low repeatability and heritability (estimated here by the proportion of variation due to sires). A preliminary study of 105 ewe lambs (Pinares-Patiño *et al.*, 2011b) in New Zealand (NZ) estimated repeatability at 0.16±0.10 and heritability at 0.30±0.26 for MY based on three of four RC chamber measurements (the first was considered part of the adaptation period). A larger follow-up study (Pinares-Patiño *et al.*, 2013) measured 1205 sheep for 2 consecutive days in the RC then another 2 consecutive days a fortnight later. A single MY measurement had estimated heritability of 0.13. Repeatability on consecutive days was 0.89, but only 0.26 for measurements 2 weeks apart, and 0.24 across years. In our study, the CV of sire variation of 2.0% for MY0 corresponds to an estimated heritability of 0.09 (calculated as: *h* ^2^=4×*V*_s_/(*V*_s_ *+V*_a_ *+V*_r_), where *V*_s_, *V*_a_ and *V*_r_ are the estimated variation for sires, animals and residuals, respectively, reported in Table 3). Thus, our results for MY0 are similar to the estimated heritability of 0.13 for MY in NZ. We did not measure consecutive days in the RC, and the last measurement in batch 5 was 14 days after the first, so our estimates of the repeatability of measurements in the same batch are not comparable to the NZ estimates of the repeatability of tests on consecutive days. However, our estimated repeatabilities of measurements in different batches (0.22 for MY0, 0.28 for MY3) are similar to the NZ estimates of 0.26 for measurements after a 2-week interval and 0.24 across years.

### Effect of feed intake

As well as confirming the results of other researchers, we gained additional insights into the key role of feed intake in the RC and its relationship with animal and sire differences in MY. For our sheep, fed at 20 g/kg LW, confinement in the RC was associated with reduced %FE, particularly for heavier animals, and especially for repeat RC tests within 2 days of each other. Significant sire variation, over and above the effect of LW, was noted in %FE for RC tests 2 and 4 days after the initial RC test. Moreover, when MY was corrected for feed intake in the previous 2 days, estimated sire variation decreased to zero.

Reductions in feed intake (kg) were also noted by Robinson *et al.* (2011) when sheep, with *ad libitum* access to feed, were confined in RC. DMP and FIC were highly correlated (*r*=0.72 to 0.75), but MY0 (calculated as emissions in the RC divided by FIC) had a strong negative correlation with FIC (*r*=−0.63). When feed intake on the day before testing was included in the calculation, the correlation (*r*=−0.19) was weaker (Robinson *et al.*, 2011).

Arthur *et al.* (2012) reported a modest positive correlation of 0.33 between feed intake and DMP of cattle, and also a negative correlation (*r*=−0.26) between feed intake and MY0. For sheep fed at 1.4*x* maintenance, Goopy *et al.* (2009) also reported a low correlation of 0.17 between FIC and DMP in the RC, with the data showing a negative correlation between FIC and MY0 (*r*=−0.58). In general, the relationship between feed intake and methane emissions when animals have *ad libitum* access to feed appears to be less consistent than under restricted feeding, leading to increased potential for negative correlations between feed intake in the RC and MY0. In the research cited above, correlations of FI with DMP ranged from 0.17 to 0.75; correlations of FI with MY0 ranged from −0.26 to −0.63.

### Avoiding biases

Although RC measurements have been considered the ‘gold standard’ for methane measurements, our results show that, despite a considerable period of adjustment on the same diet as in their RC tests, some animals tended to eat less when confined in the RC or in metabolic crates, leading to potential biases in MY0. Rather than habituating to the situation, average feed intake for the second RC test was even lower than the first, and lower still for the third. These departures from normal behaviour affected emissions, and might reduce the efficacy of genetic or phenotypic selection if the aim is to improve methane emissions under normal grazing conditions.

In our experiment, feed was removed at 1600 h on days before RC testing. This created additional variability in feed intake, which is likely to enhance the statistical significance of the effect of feed intake on the previous day. In addition, the confined conditions in the RC increased the variability of feed intake in the RC, making effects on emissions at the next RC test 2 days later easier to detect. The power to detect significant relationships (and the accuracy of estimates) depends both on the size of the variation and the confounding among the ‘*x*’ or independent variables. In the more desirable situation where animals eat the same amount of feed in the RC as on previous days, feed intake measurements on different days would be statistically confounded, making it more difficult to detect significant relationships with feed intake on specific days.

Thus, the variability in feed intake in our data helped demonstrate the desirability of measuring feed intake for at least 2 days before recording methane in the RC. Although estimates of regression coefficients can be used to derive a weighted index of feed intake (in our case, 0.509×FIC+0.342×FIP+0.148×FIP2), a simpler approach is to fit separate covariates for the relationship with feed intake on different days. This approach can help avoid biases related to different effects for different diets or management conditions, and allow for finer-scale adjustments, for example, the effect of feed intake in 6- or 12-h periods before measurement. Our results suggest that simply dividing emissions by feed intake in the RC can result in a negative phenotypic correlation with feed intake. Similar effects for genetic correlations might lead to increased overall feed intake of the selected animals, potentially increasing feed costs, or reducing efficiency relative to production.

We observed significant differences between test batches for MY0, MY3 and DMP (adjusted for LW or feed intake in the RC and 2 previous days). The design of RC experiments comparing results for animals from different test batches should therefore include repeat tests of some animals to facilitate the comparison of results from different batches.

In many situations, even when animals are housed in identical conditions, feed intake can have modest repeatability, for example, for steers being finished for Korean or Japanese markets, Robinson and Oddy (2001) reported average correlations of 0.19 for daily feed intake on consecutive days and 0.31 for intake on non-adjacent days in the same week. Accounting for feed intake on previous days should therefore improve accuracy, irrespective of whether the variation in feed intake is completely random, or affected by systematic variation related to test conditions. This is illustrated by the many significant factors identified in this study, including, test day, chamber, chamber×batch, LW and twins reared as singles. In our study, most of these effects were not evident for MY0, but became statistically significant for MY3, which had less than half the residual variation of MY0.

### Permanent animal effects

Even though no differences relating to sires could be detected, phenotypic animal differences were apparent – the SD of animal effects represented about 5% of the mean. The significant relationship of MY3 with LW (*P*=0.022), and the fact that twins reared as singles had higher MY3 than expected for their feed intake or LW (*P*<0.01), are examples of how environmental factors can influence MY. Sheep born as twins tend to have lower birthweights than singletons, but can exhibit some compensatory gain when reared as singles compared with their twin-reared flockmates.

Detailed investigation into the gut kinetics of 10 highest and 10 lowest MY sheep retested in batch 5 revealed that the low emitters had shorter mean particulate retention time (1.11 *v*. 1.34 days, *P*<0.002), less rumen particulate contents (*P*=0.007) and a smaller rumen volume (*P*=0.048; Goopy *et al.*, 2014a). This supports the finding of Pinares-Patiño *et al.* (2011a) and Barnett *et al.* (2012) that shorter retention times in the rumen are phenotypically associated with lower MY.

The lack of evidence for differences in MY related to the animals’ sires, despite significant animal effects for MY3 and MY0 (and indeed significant sire variation for %FE on days 2 and 4 of RC testing) remains a matter for speculation. Although the 160 sheep originated from the SheepGENOMICS flock, which was created to provide a high level of genetic diversity to facilitate gene discovery (White *et al.*, 2012), the lack of sire variation for MY may simply reflect the small numbers of sires and animals tested.

In our experiment, batch (representing time of year and associated environmental conditions) was highly significant, with the June/July batch having 8% greater MY3 and 11% greater DMP adjusted for LW than the February/March batch. It is not known if this difference was related to diet, condition score of the animals or environmental factors such as temperature or season. There is some evidence that both diet (Torok *et al.*, 2011) and temperature (Kennedy *et al.*, 1982 ) can affect rumen microbial composition and MY, potentially altering the ranking of an animal’s emissions over the short to medium term. It is possible that diet might also have longer term or permanent environmental effects on the variation in rumen flora that influence methane emissions. If so, any variation due to sire effects, especially if expressed only in certain conditions, could be difficult to detect and exploit. Rumen volume also changes with feeding level (Purser and Moir, 1966), and has been observed to increase by 20% to 34% in the 1^st^ hour after feeding (Stewart *et al.*, 1958). Possibly, environmental factors such as amount and type of feed available to a developing animal could lead to variation rumen characteristics that might show up as permanent environmental effects of the animal.

### Genetic improvement

The absence of differences between sires for the repeated measures of MY3 in this study of 160 offspring of 20 sires does not necessarily imply that methane emissions per unit of production cannot be improved. Results from tests involving more animals, or under different conditions, for example, when animals have *ad libitum* access to food, might be more heritable. In our data set, greater reductions in feed intake were noted for the second and subsequent RC tests, suggesting that biases might be reduced by using of a single RC test, or allowing more time between tests. The estimated sire variation for the 1^st^ day of RC testing was consistent with other estimates for MY, but the large SE precludes any firm conclusions from being drawn from the limited information available.

In general, genetic improvement from traits with low heritability will require large numbers of animals to be tested (Falconer *et al.*, 1996). The development of PACs to measure methane emissions of grazing sheep offers the opportunity for a relatively inexpensive 1-h test of animals under conditions similar to those in which they will be reared. Similar systems have been used for cattle (Turner and Thornton, 1966). O’Kelly and Spiers (1992) questioned the wisdom of extrapolating measurements under highly standardised, controlled conditions, to the free-ranging situations of most livestock farming system. As well as restrictions in movement and the reduced feed intake noted in the RC in this study compared with the individual pens to which they had been adapted, animals enclosed in a chamber lack the opportunity to select their diet and interact with their peers or the natural environment.

Purvis *et al.* (2013) expressed similar concerns for beef cattle, noting that feed intake is usually conducted in a feedlot environment where animals in group pens are fed diets of very different composition and availability to the pasture swards representing the normal production system in Australia, but that novel phenotyping techniques such as miniaturised wireless sensors and data capture systems now offer a way to study livestock in the commercial production environment.

The system of measuring methane emissions of grazing animals in PACs was developed by Goopy *et al.* (2014b). A genetic analysis of 2600 1-h PAC measurements from the Australian Information Nucleus Flock reported significant sire differences in methane emissions adjusted for LW as a proxy for production (Robinson *et al.*, 2014). Although the estimated heritability of a single 1-h measurement was relatively low (11.7%), the mean of two measurements had a higher estimated heritability of 18.6%. Further work will be required to determine the relationships between emissions of the same animal at pasture and in the RC, and also at different stages of life, as well as investigate potential biases (e.g. differences in diurnal grazing patterns) in PAC measurements, in order to better understand these relationships and determine the best way of reducing total methane emissions of Australian livestock without detrimentally affecting production.
